# Enhancing urban air quality prediction using time-based-spatial forecasting framework

**DOI:** 10.1038/s41598-024-83248-z

**Published:** 2025-02-03

**Authors:** Shrikar Jayaraman, Nathezhtha T, Abirami S, Sakthivel G

**Affiliations:** Vellore Institute of Technology Chennai, Chennai, India

**Keywords:** AQI, TBS, CNN, ARIMA, Spatial characteristics, Forecasting, Temporal dependencies, Climate sciences, Environmental sciences

## Abstract

Air quality forecasting plays a pivotal role in environmental management, public health and urban planning. This research presents a comprehensive approach for forecasting the Air Quality Index (AQI). The proposed Time-Based-Spatial (TBS) forecasting framework is integrated with spatial and temporal information using machine learning techniques on data collected from a wide range of cities. The TBS employs Convolutional Neural Networks (CNNs) to capture spatial dependencies based on normalized latitude and longitude coordinates of the cities. Simultaneously, time series model, specifically the ARIMA (AutoRegressive Integrated Moving Average) was employed to capture temporal dependencies using pollutant concentration readings over time. The dataset included information such as date, time, pollutant concentrations and AQI was further preprocessed and divided into training and testing sets. The CNN was configured to utilize the normalized latitude and longitude grid, while the ARIMA model concurrently processed the pollutant concentrations. The model was trained on the training dataset, and a 6 hour forecast is generated for each test instance. The outcomes demonstrate the TBS model’s ability to accurately predict AQI values. The integration of CNNs and time series model allowed for an clearer and deeper understanding of geographical and pollutant concentration factors that contribute to air quality variations.

## Introduction

Air Quality Index stands at the forefront of environmental concerns, representing a crucial measure of air pollution’s and its impact on human health along with the environment. The relentless urbanization and industrialization of our societies have led to a surge in air pollution levels, jeopardizing the well-being of communities worldwide. The consequences of poor air quality are profound, contributing to respiratory ailments^1^, cardiovascular diseases, and a reason of other health complications. Moreover, the long-term ecological impact extends to vegetation, wildlife, and overall environmental sustainability. To overcome these challenges, there is a need for accurate and timely AQI forecasting. An effective forecasting model serves as an invaluable tool for policymakers, urban planners, and public health officials, offering the ability to anticipate and mitigate the adverse effects of air pollution. By predicting AQI trends, cities can implement proactive measures to protect their residents, ranging from traffic management^2^ to industrial regulations.While the importance of AQI forecasting is evident, the existing methods often fall short of providing a holistic solution. The gaps in research include:

- The existing research lacks a comprehensive model that adeptly combines spatial and time-based dependencies in air quality forecasting. Many methodologies tend to focus exclusively on either spatial^3,4^ or temporal aspects^5^, overlooking the intricate interplay between geographic locations and pollutant concentrations over time.
- A significant gap exists in the ability of current forecasting models to generalize across diverse city profiles^6,7^. The variations in urban structures, environmental factors, and pollutant sources among different cities pose a challenge for existing methodologies to provide accurate and applicable predictions universally.
- The demand for real-time air quality forecasting is on the rise, yet the current research landscape lacks models capable of swift and accurate real-time implementation and deployment^8,9^. There is a need for methodologies that can promptly identify and respond to dynamic air quality changes, surpassing the capabilities of traditional forecasting methods.
- Current methodologies face challenges in effectively handling data imbalances and accounting for the diverse geographical characteristics present in air quality datasets. The uneven distribution of data points across pollutant concentrations and geographical regions requires tailored approaches to ensure accurate and unbiased forecasting results.
- The research addresses the gaps in current research methodologies and proposes a novel Time Based-Spatial forecasting framework for AQI hereon referred to as *TBS*. The proposed TBS framework seeks to bridge the gap between spatial and temporal considerations, by utilizing Convolutional Neural Networks (CNNs)^10^ for spatial analysis and time series models, and usesAutoRegressive Integrated Moving Average (ARIMA) for temporal dependencies. By integrating these techniques, the TBS aims to create a comprehensive model capable of delivering accurate and nuanced AQI forecasts. The Time-Based-Spatial (TBS) method leverages the centralized data collection infrastructure of the Central Pollution Control Board (CPCB) to enable real-time forecasting of air quality across the country. The CPCB monitors air quality data from numerous stations nationwide, ensuring a consistent and timely flow of information. The methodology used in this study enables live mining and processing of pollutant data directly from the CPCB’s centralized repository. By integrating this live data feed into the forecasting model, the TBS framework can dynamically update predictions, providing real-time AQI forecasts. This capability is critical for rapidly changing urban environments, where real-time predictions allow for actionable insights, such as traffic management or public health advisories, to mitigate the adverse effects of air pollution. The TBS framework addresses data imbalances by utilizing a highly diversified dataset encompassing AQI data from 22 cities across urban, suburban, and rural areas in India. This diversity captures a wide range of environmental conditions, levels of industrialization, and geographic features, ensuring balanced representation of air quality patterns. By normalizing pollutant concentrations and embedding spatial coordinates, the framework reduces biases, enabling the model to generalize effectively across diverse locations and deliver accurate AQI forecasts.

The motivation behind TBS:

- This research focuses on analyzing air quality dynamics over time by utilizing diverse geographical dataset from the Central Pollution Control Board (CPCB) of India, covering 22 major cities. The air pollution patterns are examined using temporal records and pollutant features aiding environmental management and public health initiatives.
- The project tackles the multifaceted challenges of air quality forecasting by leveraging the dataset’s diversity, which spans urban, suburban, and rural areas across India. Incorporating spatial and temporal variations, the research aims to develop robust forecasting models capable of capturing seasonal trends and long-term fluctuations, enhancing the accuracy of predictions and informing environmental policies.
- To develop a hybrid model of Convolutional Neural Network (CNN) and AutoRegressive Integrated Moving Average (ARIMA), the project adopts an innovative approach to air quality forecasting. By combining spatial and temporal dependencies through weighted averaging, the proposed framework promises accurate and actionable forecasts, contributing to advancements in environmental monitoring and public health interventions.
- A novel architecture is proposed that combines spatial and temporal factors for accurate AQI prediction. The model is validated using diverse urban data from 22 cities and evaluated comprehensively over a 6-h time window, demonstrating its robustness and practical applicability.

The paper is organized as follows section “Prior research on air quality index” discuss the prior research on Air Quality Index, section “Methodology” explains about the working of proposed TBS, followed by result and discussion in section “Results and inferences”. Finally, the paper is concluded in section “Conclusion and future work”.

## Prior research on air quality index

The Air Quality Index (AQI) plays a major role in indicating the level of air pollutants, which has an impact on environmental protection. This section explores various techniques and methodologies employed for AQI. Kumar K et al.^11^ emphasizes the transformative potential of machine learning techniques in contrast to traditional methods for predicting air quality. The data on air pollution from 23 Indian cities spanning six years were used, employing a meticulous preprocessing approach and correlation analysis to identify key features influencing air quality. The author utilized the machine learning models like Gaussian Naive Bayes, Support Vector Machine, and XGBoost. These models were compared using common metrics, in which the Support Vector Machine model performs the worst and the Gaussian Naive Bayes model performs the best. With the highest linearity between the predicted and actual data, the XGBoost model notably performs better than the other models.

Furthermore, air pollution’s severe health implications necessitate effective forecasting mechanisms for timely intervention by authorities. Machine Learning, especially Deep Learning models, has emerged as a prominent solution for air quality prediction with multiple contributions entering the field. Manuel Méndez et al.^12^ provide a comprehensive review of significant contributions in air quality forecasting from 2011 to 2021. The survey, which comprises 155 carefully chosen study, groups contributions according to evaluation metrics, machine learning models, predicted values, predictor variables, and geographic distribution. On the other hand, Iskandaryan et al.^13^ focus on the challenges of air quality monitoring and forecasting. It highlights the increasing reliance on machine learning and deep learning methods due to their effectiveness in processing multidimensional information. For the purpose of predicting air quality, the Attention Temporal Graph Convolutional Network method integrates Attention, a Gated Recurrent Unit, and a Graph Convolutional Network. Data on air quality, weather, and traffic from the city of Madrid for the months of January through June 2019 and January through June 2022 were used to test the suggested approach. When compared to the reference models (Temporal Graph Convolutional Network, Long Short-Term Memory, and Gated Recurrent Unit), the results validated the benefits of the suggested model.

Chen et al.^14^ address the challenge of overfitting in air quality prediction algorithms based on single models. The proposed approach integrates Seq2Seq, LSTM with attention mechanism, and XGBoosting to enhance prediction accuracy. The Seq2Seq model establishes a single-factor prediction, treating each air quality component as independent time series data. Subsequently, the LSTM^15^ model incorporates multi-factors, considering data from neighboring stations and meteorological variables. Finally, XGBoosting combines the strengths of both models, providing more accurate predictions. Ganesh et al.^16^ focus on forecasting air quality index (AQI) in specific areas. The study uses linear models like multiple linear regression with gradient descent variants and regression models like Support Vector Regression (SVR). The AQI predictions are based on pollutant concentrations of NO_2_, CO, O_3_, PM2.5, PM10, and SO_2_. Notably, the Support Vector Regression model demonstrated superior performance across investigated quality measures.

Chandar et al.^17^ address the challenge of predicting short-term Air Quality Index (AQI) for San Francisco, CA. Using ten years of historical AQI data, the study focuses on primary pollutants such as CO2, CO, NOx, PM, and SO2. Trends, levels, cyclicity, and seasonality are analyzed to forecast the next seven-day and 30-day window periods. Metrics like Mean Absolute Error (MAE) and Root Mean Square Error (RMSE) are used in the deployment and evaluation of machine learning and deep learning models, such as Random Forest, Support Vector Regression, XGBoost, Neural Network, and Long Short-Term Memory. Song et al.^18^ proposed a Multi-Pollutant Space–Time Learning Network (Multi-AP) estimates hourly, grid-level air pollution concentrations using data from monitoring stations and urban features like land use, traffic, and weather. The model integrates multiple pollutants in a single framework, reducing computation time by two-thirds compared to pollutant-specific methods. Meteorological data were found to be the most influential input. This approach supports efficient air pollution exposure estimation for urban health management. The Deep-MAPS framework leverages machine learning with mobile and fixed air quality sensors to achieve high-resolution. Urban big data is integrated to uncover pollution causes, supporting evidence-based urban management. Compared to fixed-sensor-based ubiquitous sensing, Deep-MAPS reduce hardware investment by up to 90%. This approach enables efficient and cost-effective monitoring of urban air quality with enhanced spatiotemporal accuracy^19^. NetGBM accurately predicts ozone levels across China at daily, weekly, and monthly scales, using land use and meteorological data for high-resolution mapping. It identifies temperature as a key factor and highlights urban areas are as having the highest ozone-attributed mortality. The model excels in regions with sparse monitoring, offering robust ozone modeling and health risk insights^20^.

Ning Jin et al. proposed a Multiple Nested Long Short Term Memory Networks (MTMC-NLSTM)^5^. The difficulties of precisely predicting the Air Quality Index (AQI) with current machine learning (ML) and deep learning (DL) technologies are covered in the paper. The Multiple Nested Long Short Term Memory Networks (MTMC-NLSTM), a novel DL framework, is introduced in the proposed methodology to address the challenges of highly volatile AQI pattern changes and intercorrelation between various AQI components. The proposed model is informed by federated learning, aiming to enhance the accuracy of AQI forecasting.

In selecting ARIMA (AutoRegressive Integrated Moving Average) over other deep learning forecasting models for the proposed project, several key factors have been considered. Firstly, ARIMA models offer a level of *interpretability* that is often lacking in more complex deep learning architectures. With ARIMA, the underlying components—autoregressive, differencing, and moving average—provide clear interpretations, making it easier to understand the patterns within the data and the factors influencing forecasted values. This transparency is particularly beneficial in forecasting a measure such as AQI, where the interpretation of air quality index (AQI) forecasting is crucial for decision-making. Moreover, ARIMA models are well-suited for datasets with moderate sizes, such as the AQI data collected from various locations. While deep learning models like Long-Short-Term-Memory networks (LSTM) may offer powerful capabilities, they typically require larger datasets to train effectively and may struggle with smaller datasets. ARIMA’s *efficiency with moderate-sized data* makes it a practical choice for this particular forecasting task.

Another advantage of ARIMA is its ability to *capture temporal patterns inherent in time series data*, such as seasonality and trends. Given that our project involves forecasting AQI values over time, ARIMA’s capacity to model these temporal patterns is essential. Deep learning models may not perform as well in this regard, especially in capturing long-term dependencies and subtle temporal nuances present in the data. Additionally, ARIMA serves as a strong baseline for time series forecasting tasks. By establishing a benchmark performance with ARIMA, we can effectively evaluate the improvement gained from more complex deep learning models. This approach ensures that any enhancements achieved through deep learning are significant and not merely due to model complexity. Finally, ARIMA models are *computationally efficient* compared to deep learning architectures. Since the proposed project involves forecasting AQI values for multiple locations, the computational resources and training time required for ARIMA are minimized, making it more practical for real-time or near-real-time forecasting applications.

## Methodology

Analyzing the air dynamics has significant challenges, Time-Based-Spatial (TBS) is proposed for AQI forecasting, the data is sourced from the Central Pollution Control Board of India encompassing 22 major cities. The dataset includes temporal and spatial features such as timestamped records, geographic coordinates, pollutant concentrations and Air Quality Index (AQI). This diversity allows for comprehensive exploration of seasonal variations and long-term trends in air quality, crucial for developing robust forecasting models. The dataset’s breadth across urban, suburban, and rural areas offers insights into complex pollutant interactions and their environmental impacts, supporting enhanced environmental monitoring and public health initiatives.

### Data collection and source

The dataset utilized in this study was sourced from the Central Pollution Control Board (CPCB) of India, a regulatory authority that monitors and manages environmental pollution. The dataset spans across 22 major cities in India, encompassing diverse geographical and environmental conditions^21^. The cities included in the dataset are Thiruvananthapuram, Chennai, Bangalore, Hyderabad, Visakhapatnam, Pune, Mumbai, Bhubaneshwar, Raipur, Bhopal, Ahmedabad, Udaipur, Kanpur, Lucknow, Varanasi, Patna, Delhi, Durgapur, Kolkata, Aizawl, Chandigarh, and Srinagar. This method of collection further enables live mining and processing of pollutant data directly from the central pollution control board, this providing real time forecast of AQI across the country.

### Feature selection

The collected dataset is rich in features, capturing temporal and spatial aspects of air quality. Key features include timestamped records (Date and Time), geographical coordinates (latitude and longitude) of the individual cities, concentrations of various pollutants (PM2.5 (Particulate Matter 2.5 µm), PM10 (Particulate Matter 10 µm), NO2 (Nitrogen Dioxide), SO2 (Sulfur Dioxide), CO (Carbon Monoxide), O3 (Ozone), NH3 (Ammonia), and the AQI. The timestamped data allows for a comprehensive exploration of air quality dynamics over time, while the spatial information provides insights into variations across the geographical landscape of the country.

**Fig. 1:**
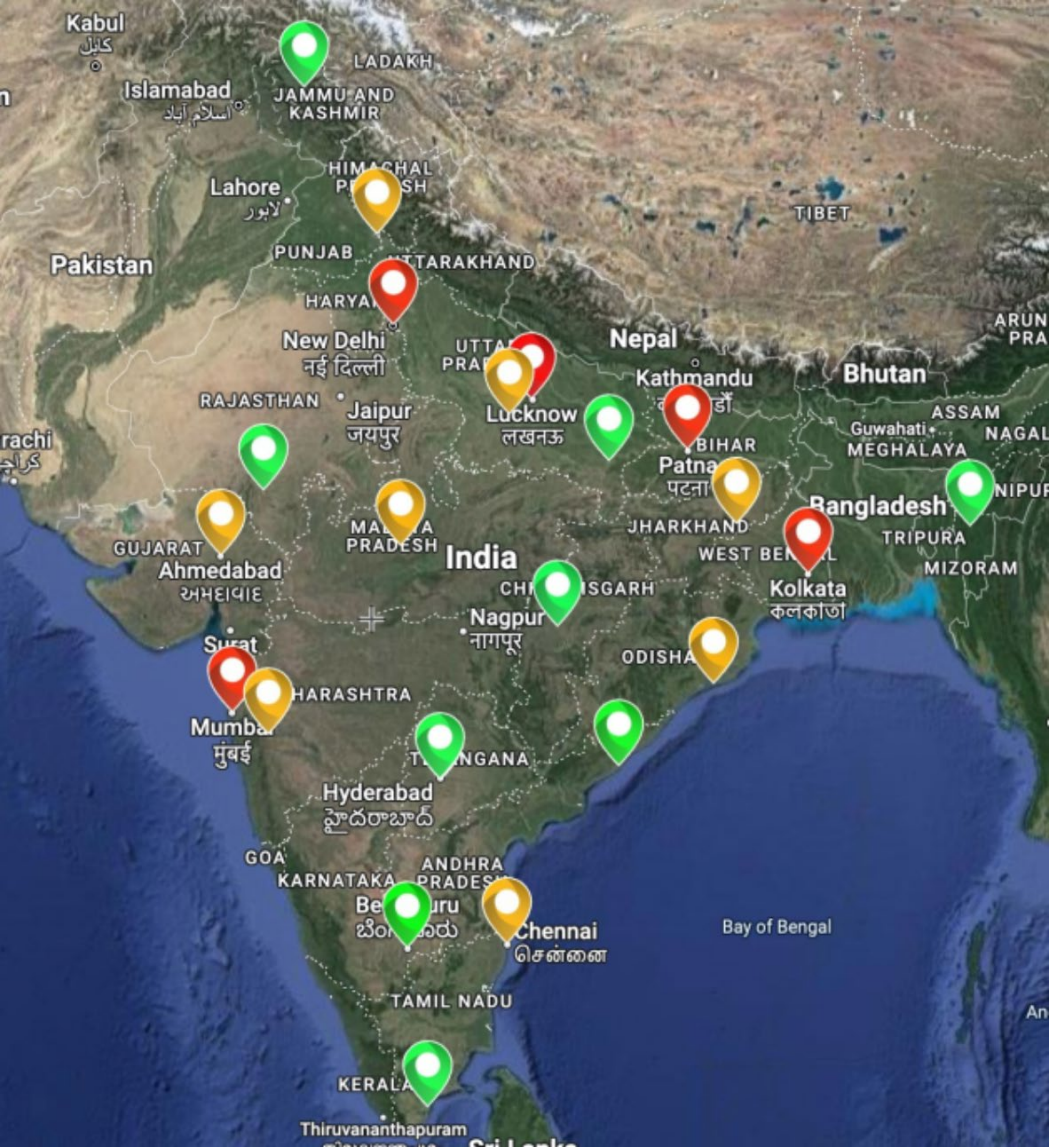
Distribution of Selected Cities, where Red represents city with bad AQI, Green represents city with good AQI, and Orange represents cities with moderate AQI^22^.

The collected dataset is highly diversified, encompassing air quality index (AQI) data from a wide range of geographic locations, each with its own unique characteristics and environmental factors. The dataset includes observations from urban, suburban, and rural areas, representing various levels of industrialization, population density, and geographic features. This diversity is essential for capturing the full spectrum of air quality conditions and understanding the complex interactions between different pollutants and environmental variables and helps mitigate the problem of data imbalances. Furthermore, the dataset covers a broad temporal range, spanning an entire year and multiple seasons. This temporal diversity allows for capturing seasonal variations in air quality, as well as long-term trends and periodic fluctuations that may influence AQI values over time. By incorporating data from different time periods, the model can account for changes in environmental regulations, urban development, and other factors that may impact air quality. Additionally, the dataset includes measurements of various air pollutants such as particulate matter (PM2.5 and PM10), nitrogen dioxide (NO2), sulfur dioxide (SO2), carbon monoxide (CO), ozone (O3), and ammonia (NH3). Each pollutant has its own sources, emission patterns, and health implications, contributing to the overall complexity of the dataset. This diversity of pollutants allows the analysis of the multifaceted nature of air pollution and development of comprehensive forecasting models that account for the interactions between different pollutants. Overall, the diversity of the obtained dataset is a key strength that enables the development of robust and generalizable forecasting models capable of accurately predicting air quality across diverse geographic regions and temporal scales. By leveraging the rich and varied information contained in the dataset, the complex challenges associated with air quality forecasting can be addressed and contributions can be made to improved environmental monitoring and public health initiatives. The following Fig. 1 shows the distribution of the cities across the country.

### Data preprocessing

Prior to analysis, several preprocessing steps were undertaken to ensure data integrity and uniformity. The original data obtained from the CPCB only accounted for Date, Time, PM2.5 (Particulate Matter 2.5 µm), PM10 (Particulate Matter 10 µm), NO2 (Nitrogen Dioxide), SO2 (Sulfur Dioxide), CO (Carbon Monoxide), O3 (Ozone), NH3 (Ammonia). This meant the spatial information and the resultant AQI values had to be generated separately. Thus for each row 4 additional columns were added during preprocessing namely, Place, Embedded Latitude, Embedded Longitude, AQI.

The raw data once obtained from the CPCB was first cleaned. Missing values were addressed through median imputation, and timestamps were standardized. Following this, the Place column was inserted which contained the name of the city to which the record belonged to. Next to be added was the Geographical data.

For each city present in the dataset, the coordinates of the city (latitude and longitude) were added as a column to account for spatial information. Once this was accomplished the latitude and longitude were converted to spatial embeddings which were the normalized coordinates of the city. This process was done using Max–Min normalization.

$${X}_{Normalized}= \frac{X-{X}_{Min}}{{X}_{Max}- {X}_{Min}}$$

For Latitude (X) and Longitude (Y)

$${Latitude}_{Normalized}= \frac{Latitude-{Latitude}_{Min}}{{Latitude}_{Max}- {Latitude}_{Min}}$$

$${Longitude}_{Normalized}= \frac{Longitude-{Longitude}_{Min}}{{Longitude}_{Max}- {Longitude}_{Min}}$$

The maximum and minimum values for the normalization were obtained from the northernmost, southernmost, easternmost and westernmost coordinates of the country which were

$$lat\_max = 35.6644^\circ N$$

$$lat\_min = 6.7562^\circ N$$

$$lon\_max = 97.4166^\circ E$$

$$lat\_max = 68.4847^\circ E$$

Once all the spatial information was added, then the AQI labels for each record were added following the National Ambient Air Quality Standards (NAAQS) as shown in Table 1.

**Table 1:** NAAQS standards for PM10, PM2.5, CO and Ozone.

Indian AQI	Indian Range (24 h)						
	PM10 (ug/m^3^)	PM2.5 (ug/m^3^)	CO (mg/m^3^)	Ozone (ug/m^3^)	NO2 (ug/m^3^)	NH3 (ug/m^3^)	SO2 (mg/m^3^)
0–50	0–50	0–30	0–1.0	0–50	0–40	0–200	0–40
51–100	51–100	31–60	1.1–2.0	51–100	41–80	201–400	41–80
101–200	101–250	61–90	2.1–10	101–168	81–180	401–800	81–380
201–300	251–350	91–120	10.1–17.0	169–208	181–280	801–1200	381–800
301–400	351–430	121–250	17.1–34.0	209–748	281–400	1201–1800	801–1600
401–500	430+	250+	34+	748+	400+	1800+	1600+

With the generation of AQI for each record the preprocessing comes to an end and the dataset is ready for model building.

**Fig. 2:**
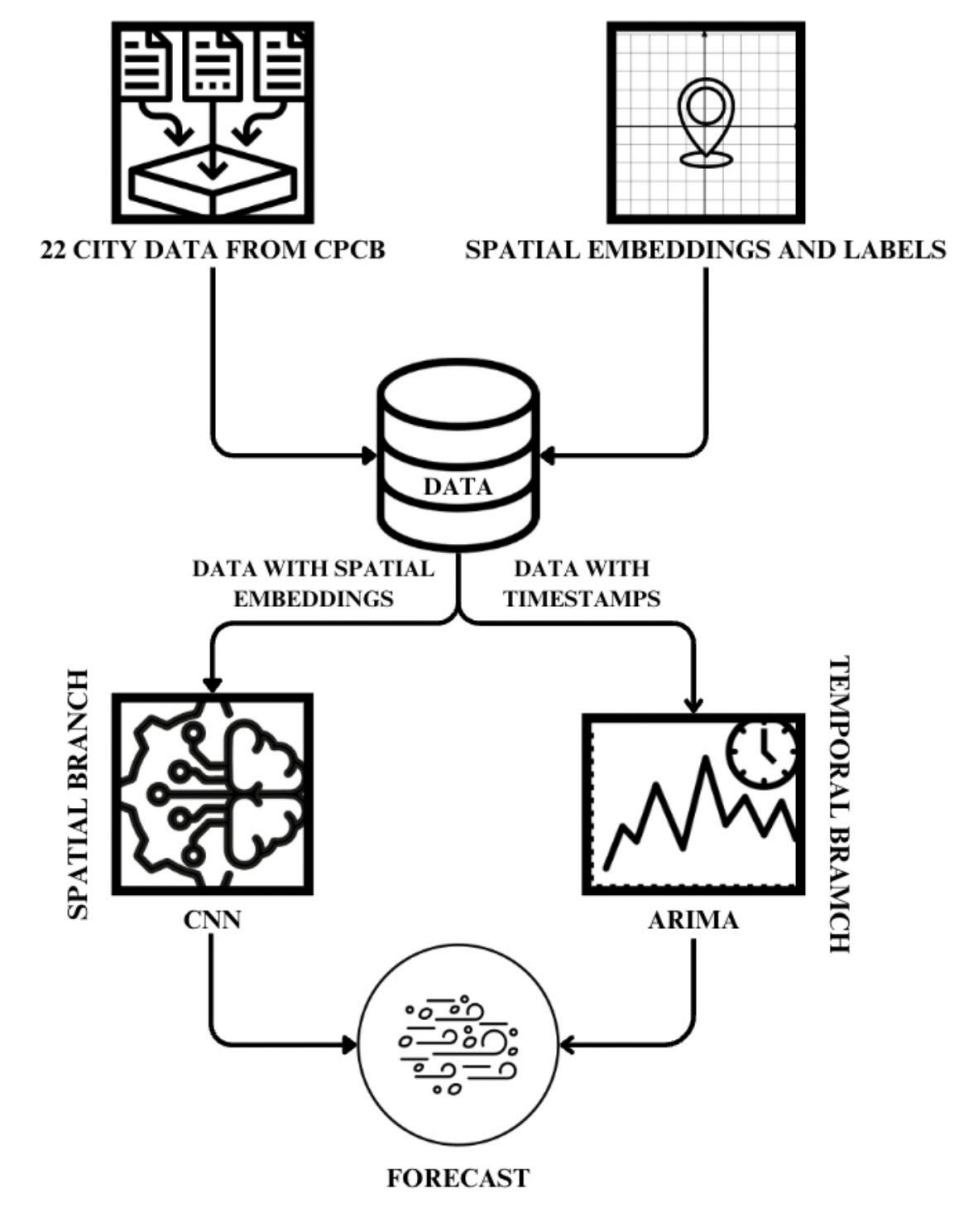
TBS Proposed Architecture.

The proposed TBS architecture is shown in Fig. 2, involved a hybrid model build consisting of a CNN^23,24^ (Convolutional Neural Network) and a Time Series model namely ARIMA^25^ model. The 2 models are trained in parallel and their outputs combined to produce the final result by means of weighted averaging. The idea was to leverage the capabilities of the CNN’s ability to process grid-like data to capture dependencies and patterns over space using the normalized latitude and longitude as inputs. Parallely, the ARIMA would work on uncovering trends and patterns of AQI over time. The resultant 6 h forecast would be of a combined nature that captures both spatial and time-based dependencies.

The 6 Hour Forecasting Window: Air Quality Index (AQI) is inherently dynamic, influenced by rapidly changing factors such as traffic emissions, industrial activity, and meteorological conditions. These fluctuations result in significant variability over short timeframes, making accurate long-term predictions challenging. While longer forecast horizons might provide broader insights, they are often accompanied by a substantial increase in error margins due to the compounding uncertainty of environmental variables over time. The 6-h forecasting window strikes a practical balance between prediction accuracy and actionable utility. It provides sufficient lead time for public health advisories, traffic management, and industrial controls while maintaining an acceptable level of prediction reliability. Extending the forecast horizon would likely result in diminished accuracy, as small deviations in pollutant concentrations or meteorological factors could lead to disproportionately large errors. This consideration aligns with the immediate need for real-time, short-term forecasting in urban environments where timely interventions can have the most significant impact.

Hence, the focus on a 6-h window ensures that the model provides actionable and reliable AQI forecasts, avoiding the trade-offs associated with longer horizons while addressing the variability inherent in AQI data.

**Fig. 3:**
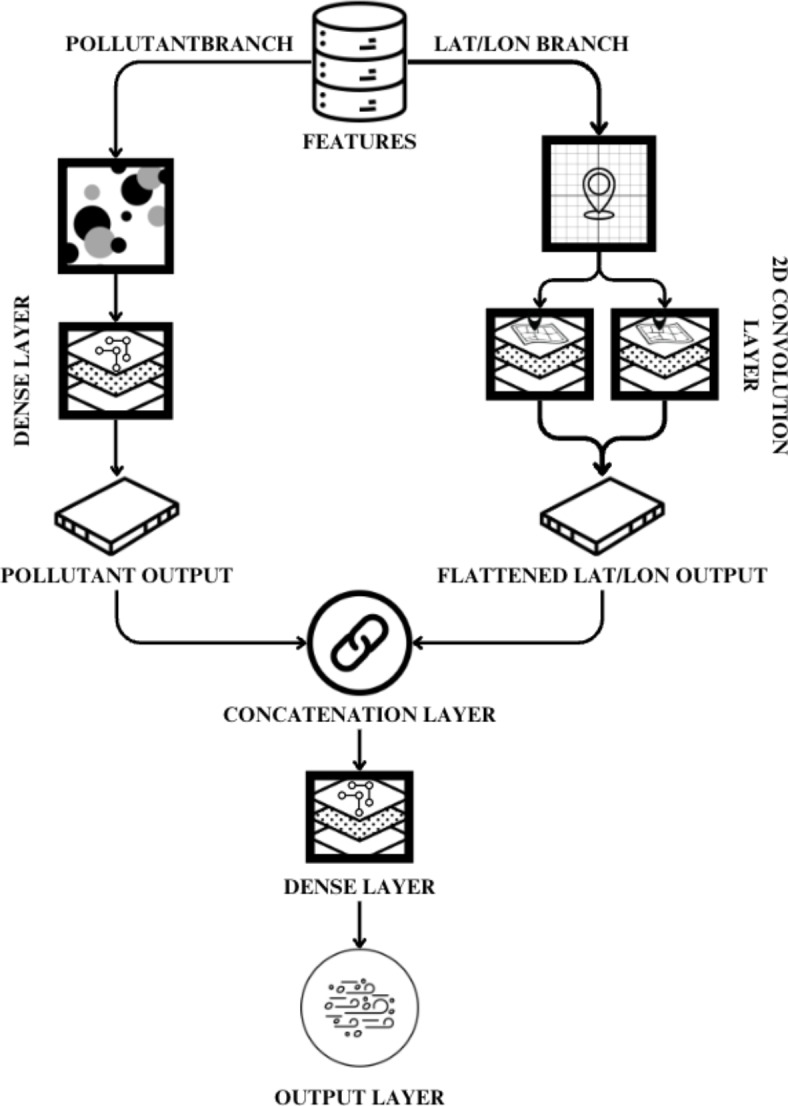
Architecture of proposed HCNN.

HCNN (Hybrid Convolutional Neural Network): The first part of the TBS consists of a CNN for which a hybrid model was proposed, the working of HCNN is given in Algorithm 1. The spatial features were extracted from latitude and longitude coordinates. The CNN architecture as shown in Fig. 3, was designed to capture spatial dependencies and pollutant variations for accurate AQI predictions. The input to the model consists of two branches: one for spatial information (latitude and longitude) and the other for pollutant concentrations. The parameters utilized in HCNN are shown in Table 2. The table outlines a neural network architecture with various layers, including input layers, convolutional, dense, and flatten layers. The model has a total of 7,974 parameters, all of which are trainable. The parameters are distributed across layers such as Conv2D (64 parameters) and Dense layers (ranging from 256 to 6 parameters). The architecture is relatively compact, with no non-trainable parameters. The spatial branch begins with a Reshape layer to adapt the input into a suitable format for convolutional operations.

**Table 2:** Parameters of Hybrid CNN.

Layer (type)	Output Shape	Param #
input_7 (InputLayer)	[(None, 2)]	0
reshape_3 (Reshape)	(None, 2, 1, 1)	0
input_8 (InputLayer)	[(None, 7)]	0
conv2d_3 (Conv2D)	(None, 2, 1, 32)	64
dense_12 (Dense)	(None, 32)	256
flatten_3 (Flatten)	(None, 64)	0
dense_13 (Dense)	(None, 32)	1056
concatenate_3 (Concatenate)	(None, 96)	0
dense_14 (Dense)	(None, 64)	6208
dense_15 (Dense)	(None, 6)	390
Total Params	7974 (31.15 KB)	
Trainable Params	7974 (31.15 KB)	
Non-trainable Params	0 (0.00 Byte)	

A 2D convolutional layer with 32 filters and a kernel size of (1, 1) was employed to extract spatial patterns. The resulting feature map was flattened to a 1D vector to be concatenated with the pollutant branch.The pollutant branch started with a dense layer of 32 neurons, followed by another dense layer with the same configuration. These layers aimed to capture intricate relationships within pollutant concentrations. Both branches were then concatenated, and the combined features were passed through a dense layer with 64 neurons and a Rectified Linear Unit (ReLU) activation function. The final output layer had six neurons to predict the AQI values for the next 6 h.

The model was compiled using the Adam optimizer and mean squared error loss function. During training, the data was split into training and validation sets, and the model underwent 15 epochs with a batch size of 32.

This neural network architecture features two input layers: one reshaped and the other directly accepting data. It includes a convolutional layer followed by dense layers for feature extraction and representation. The network combines outputs and concludes with dense layers producing a final output shape of (None, 6). It totals 7974 trainable parameters, focusing on transforming input data for specific task applications.

**Algorithm 1:**
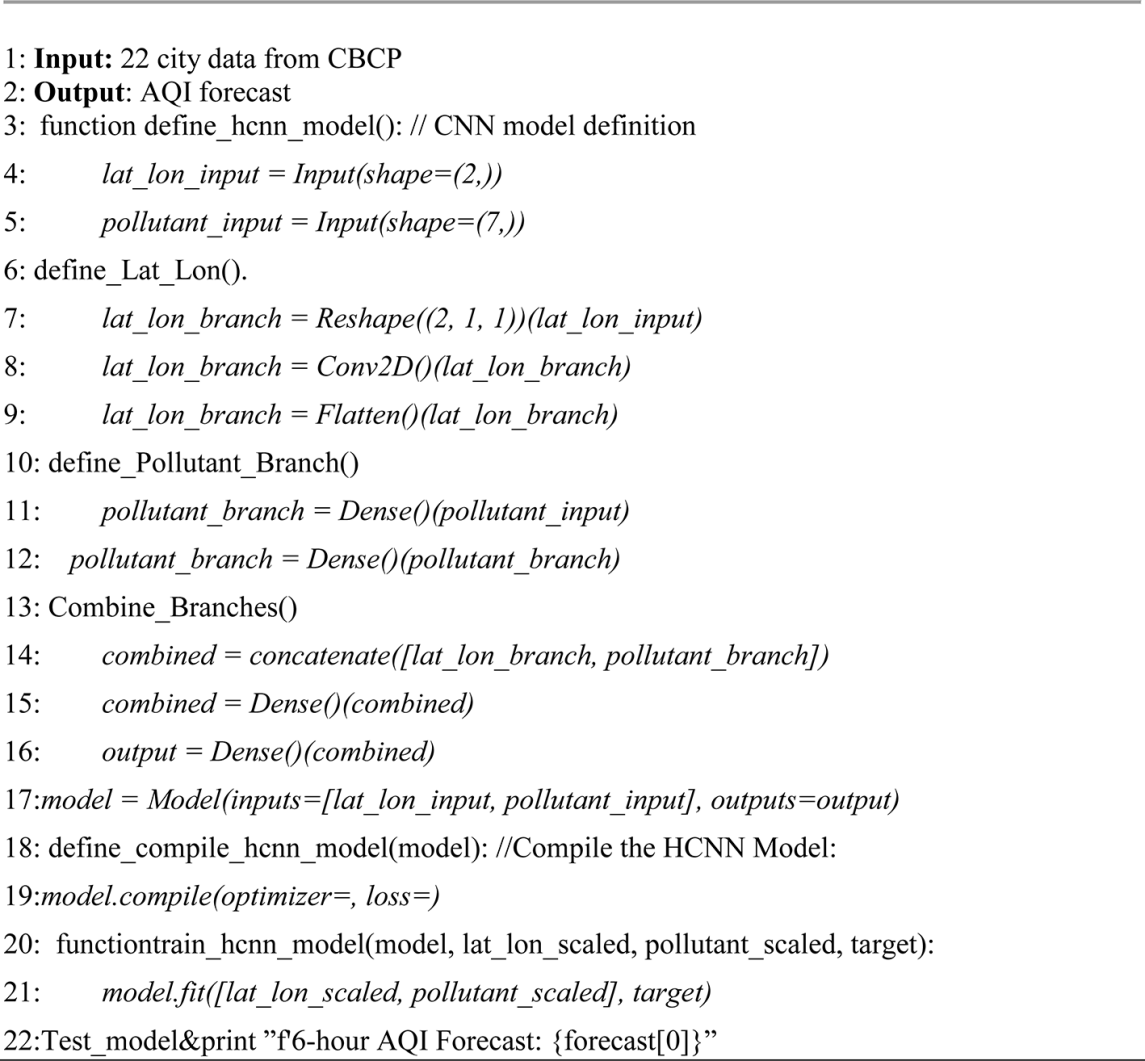
Building Hybrid CNN

**Fig. 4:**
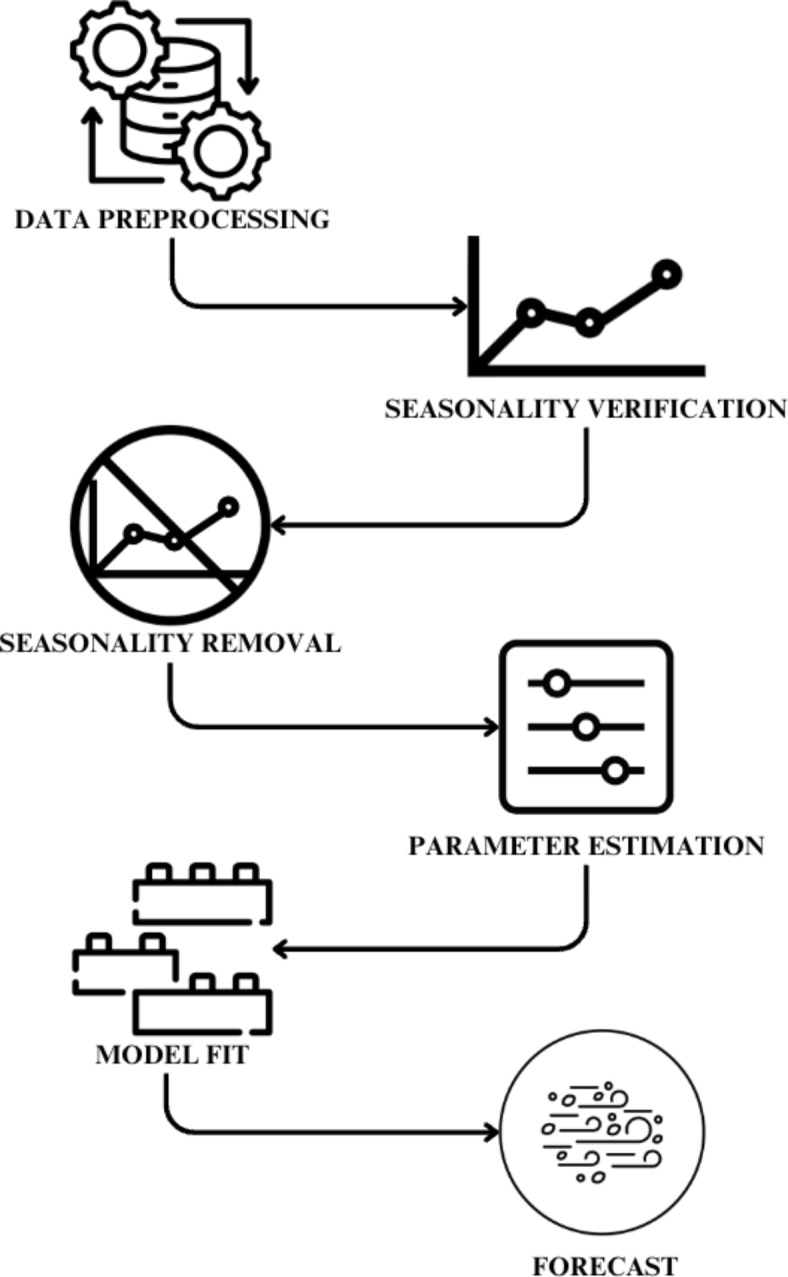
Architecture of ARIMA.

ARIMA (AutoRegressive Integrated Moving Average): The ARIMA model was employed to capture temporal patterns in Air Quality. ARIMA is a popular time series forecasting technique that uses autoregression, differencing, and moving average components to model time based dependencies. Figure 4 represents the underlying architecture of the ARIMA model.

The ARIMA model consists of three main parameters: autoregressive parameter (p), the degree of differencing (d), and the order of the moving average (q). The model aims to make predictions based on historical observations and their temporal relationships. The dataset was preprocessed to ensure stationarity, a key requirement for ARIMA modeling, through differencing operations.

To determine the optimal parameters (p, d, q) for each city’s AQI time series, a grid search approach was implemented. The grid search involved iterating over a range of values for p, d, and q to find the combination that minimizes the Root Mean Squared Error (RMSE) on the training data. This ensured that the ARIMA model was well-tailored to the specific temporal dynamics of each city.

The ARIMA models were then fitted to the training data, and the parameters yielding the lowest RMSE were chosen for each city. The fitted models were subsequently used for forecasting the AQI for the next 6 h. The forecasts provided insights into the short-term temporal variations in air quality. This was done for each of the 22 cities in our metadata.

Finally, with the outputs from both branches of the TBS loaded, the outputs were then combined using a weighted average technique in order to produce the final 6 h forecast. Thus the TBS framework leverages the two models (CNN and ARIMA) in tandem to produce a final six hour forecast for any given input instance, and the integration of the ARIMA models alongside the spatial dependencies captured by the Convolutional Neural Network (CNN) contributed to the hybrid forecasting model’s comprehensive understanding of both spatial and temporal aspects of air quality dynamics.The algorithm 2 elaborates the ARIMA model it begins by assessing the temporal pattern of a time series using the Augmented Dickey-Fuller (ADF) test^26^, checking for stationarity with a significance level of 0.05. If the series is non-stationary, indicated by a p-value above 0.05, the algorithm sets the order of differencing d to 1 to achieve stationarity. Next, using the autocorrelation function (ACF) and partial autocorrelation function (PACF), it identifies significant lags (p for AR and q for MA components) up to a specified maximum lag. Initialized variables include an empty list for storing trained ARIMA models, which are subsequently trained and evaluated for each city in the dataset to predict and print summaries of the best-fitting models. The analysis integrates both temporal and spatial features, employing a combined approach of Convolutional Neural Networks (CNN) and AutoRegressive Integrated Moving Average (ARIMA). This method utilizes temporal data such as timestamped records and historical trends in pollutant concentrations (PM2.5, PM10, NO2, NH3, SO2, CO, Ozone) to capture time-dependent patterns. Simultaneously, spatial features like geographic coordinates are processed by CNNs, enabling the model to understand and predict air quality variations across different locations. The strengths of both CNNs and ARIMA are utilized and an integrated approach is proposed that ensures a comprehensive understanding of air quality dynamics, facilitating more accurate and localized forecasts crucial for environmental monitoring and public health management.

**Algorithm 2:**
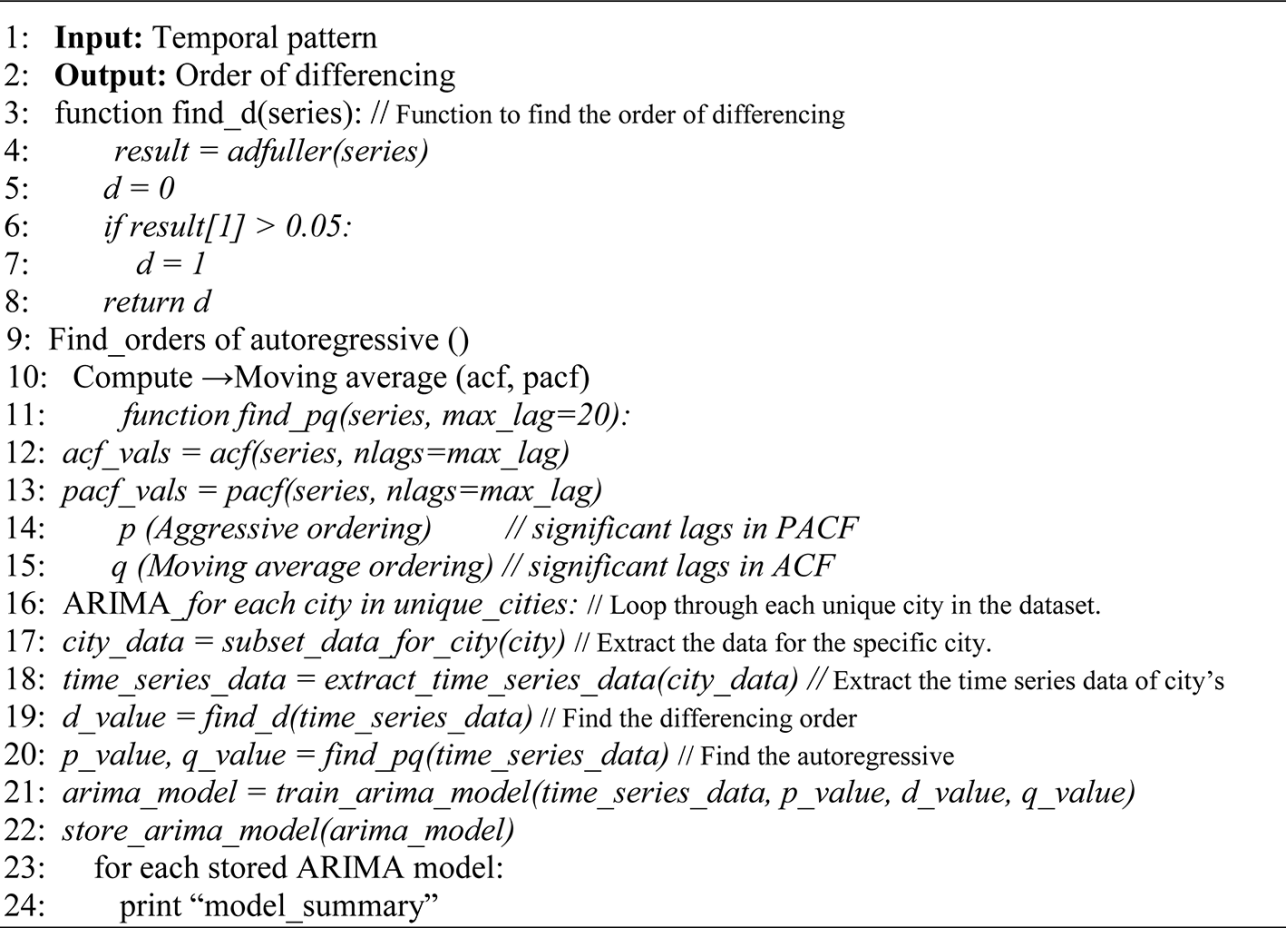
ARIMA model.

## Results and inferences

**Fig. 5:**
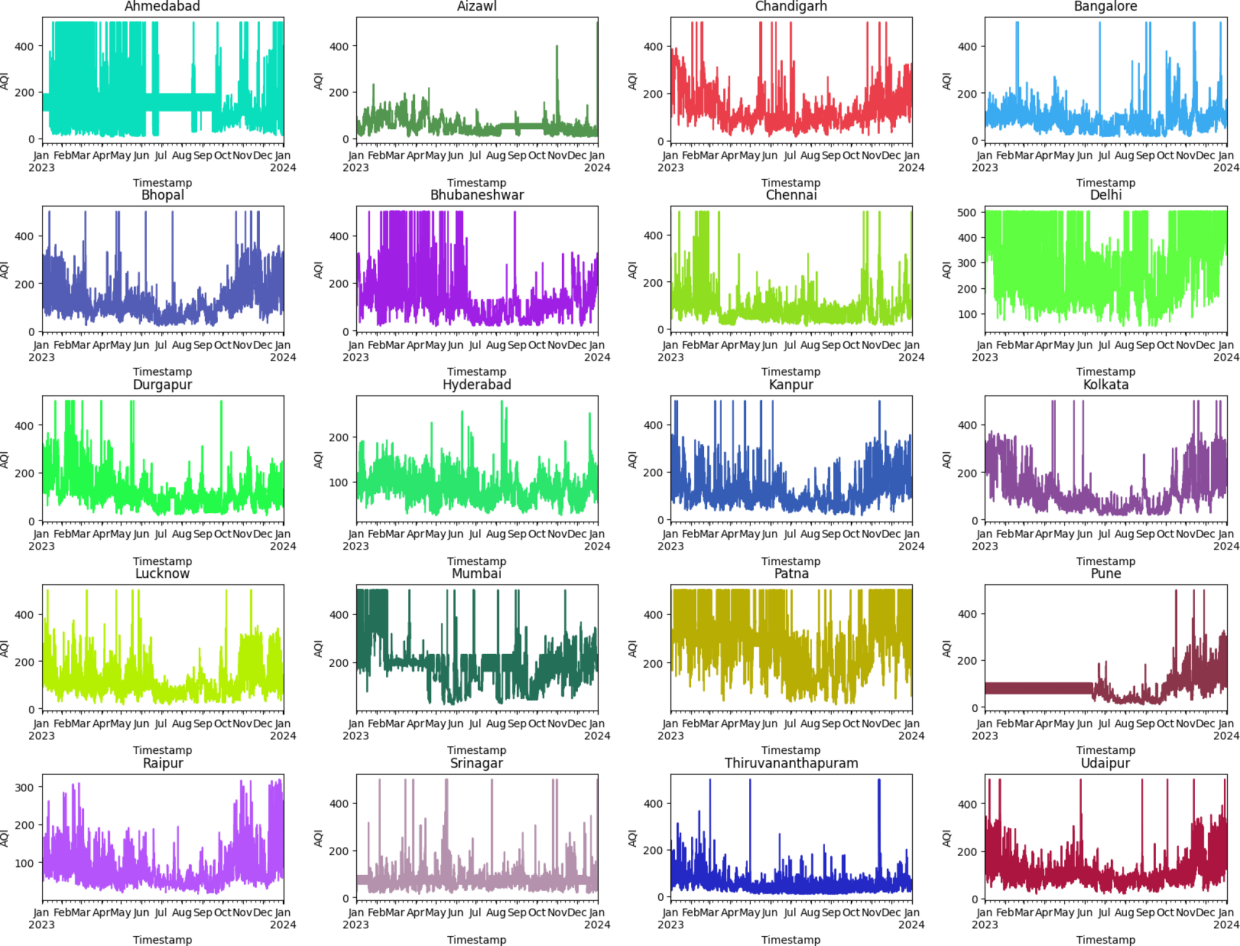
Distribution of AQI for each City.

**Fig. 6:**
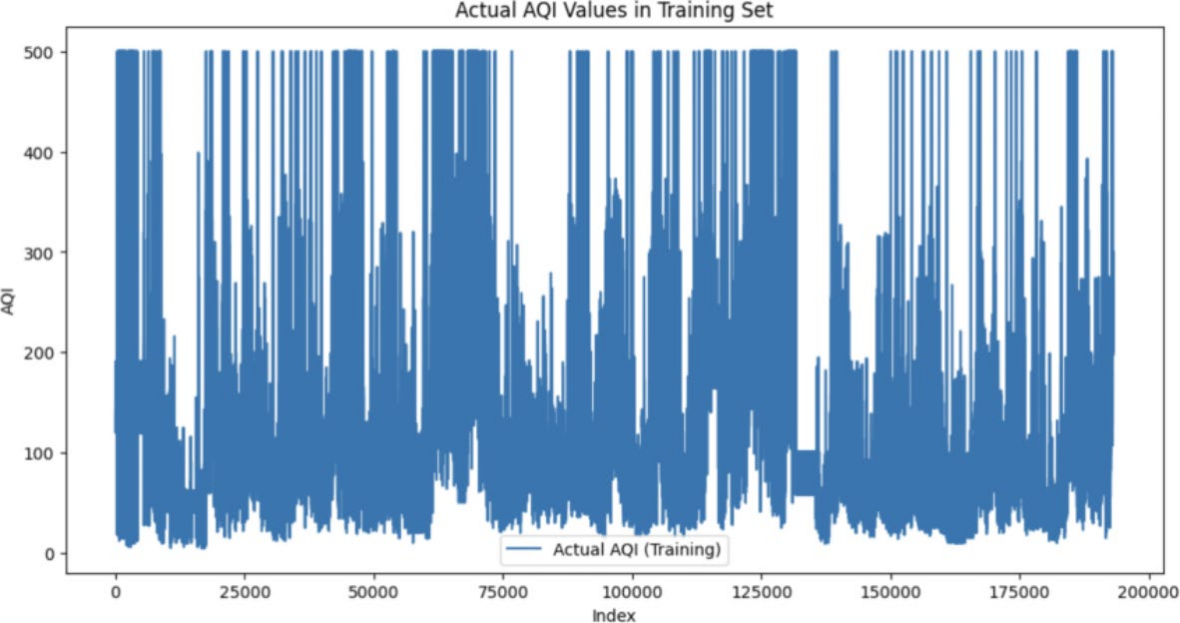
Distribution of AQI in training set.

**Fig. 7:**
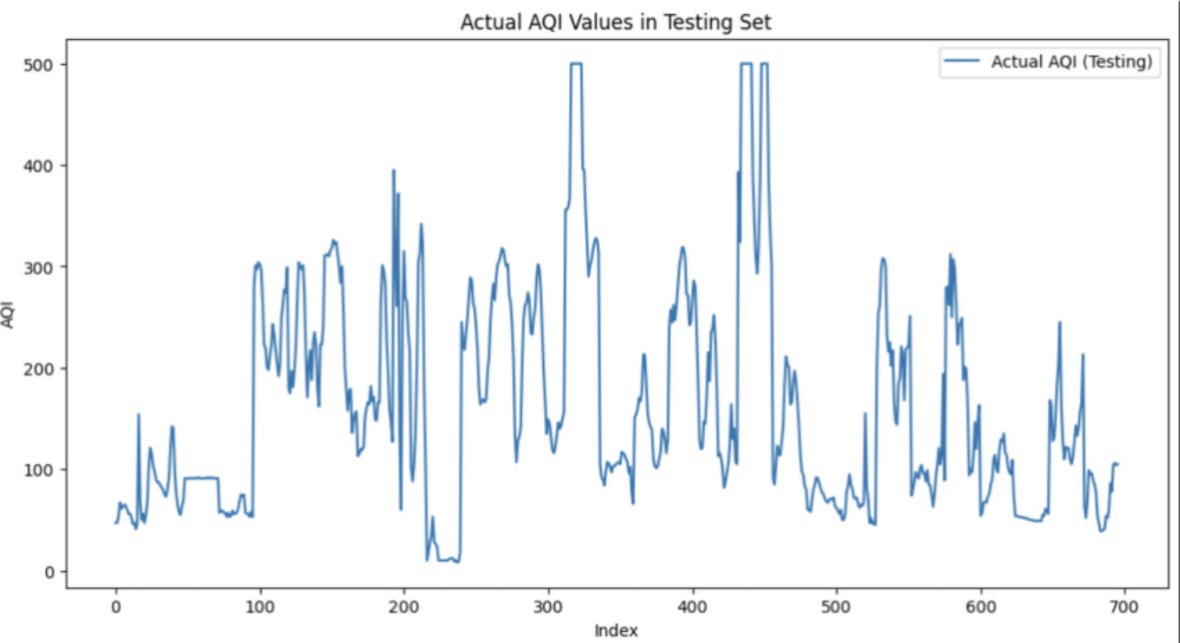
Distribution of AQI in testing set.

The hybrid forecasting model, integrating Convolutional Neural Network (CNN) for spatial dependencies and the time series model (ARIMA) for temporal patterns, exhibited promising performance in predicting the AQI. Examining the spread of AQI values further enhances our understanding of the model’s performance, the following Figs. 5, 6, 7 show the distribution of AQI across each city and across the entire training and testing sets to gain an understanding of the way the AQI is distributed:

### Standard deviation of AQI in training set: 96.61

See Fig. 6.

### Standard deviation of AQI in testing set: 108.32

The substantial standard deviation observed in both the training set (96.61) and testing set (108.32) underscores the inherent variability in the AQI data. High SD values signify a wide range of air quality conditions, likely influenced by diverse factors such as meteorological variations, industrial activities, and local emissions. This inherent variability poses a considerable challenge for accurate forecasting, as predicting extreme values becomes inherently intricate when dealing with highly fluctuating datasets.

The following key metrics were calculated to assess the model’s accuracy:

### Mean absolute error (MAE): 31.73

The MAE of 31.73 indicates that, on average, the model’s predictions deviated by approximately 31.73 units from the actual AQI values. Against the backdrop of high data variability indicated by the high SD, the Mean Absolute Error (MAE) of 31.73 emerges as a noteworthy achievement. MAE evaluates the average difference (absolute) between the forecasted and actual AQI values, indicating the model’s accuracy. The comparatively low MAE in the face of high data variability highlights the model’s resilience and adeptness in capturing diverse air quality patterns.

The model’s ability to achieve a relatively low MAE amidst challenging conditions signifies its capability to discern and forecast air quality trends effectively. This accomplishment becomes particularly crucial in urban environments characterized by dynamic and unpredictable pollution sources.

### R-squared (R2) score: 0.81

The R-squared (R2) score of 0.81 further substantiates the model’s performance. R2 determines the proportion of the dependent variable’s (AQI) variance that can be predicted by the independent variables. In the context of this study, the high R2 score indicates that a significant portion of the AQI variability is captured by the hybrid model, reinforcing its ability in modeling the complex combinations of spatial and temporal factors influencing air quality.

**Fig. 8:**
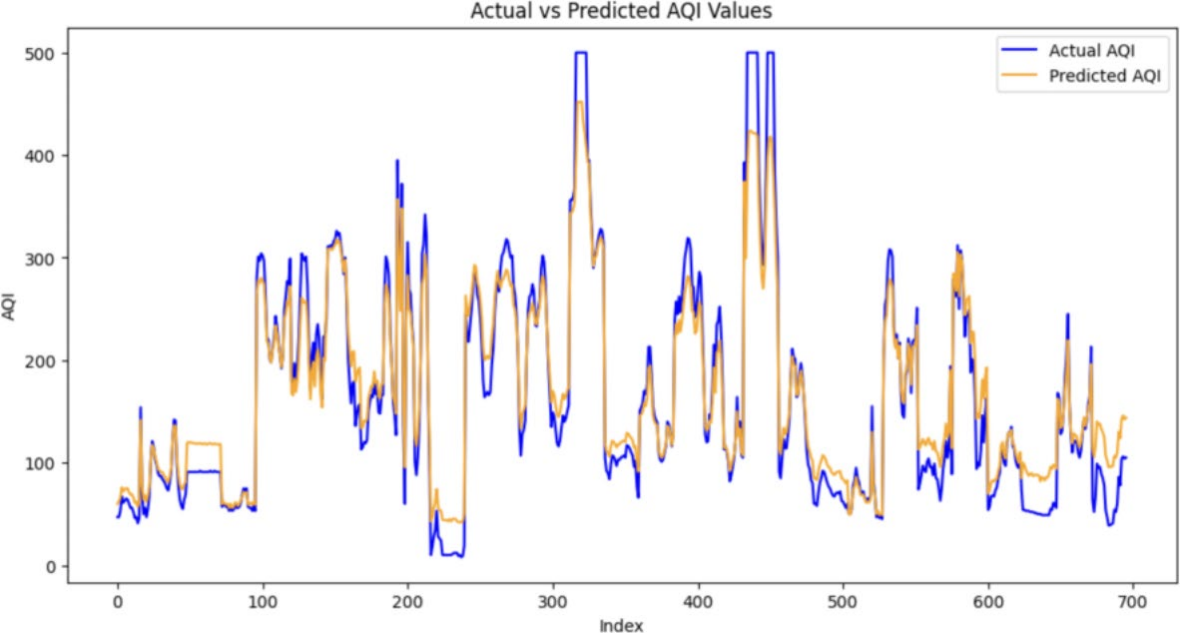
Actual vs Predicted AQI values.

To visually assess the model’s predictive capabilities, plots depicting the predicted versus actual AQI values were generated as shown in Fig. 8. These plots provide insights into how well the model captures both high and low AQI events, crucial for understanding its performance across different pollution scenarios.

**Fig. 9:**
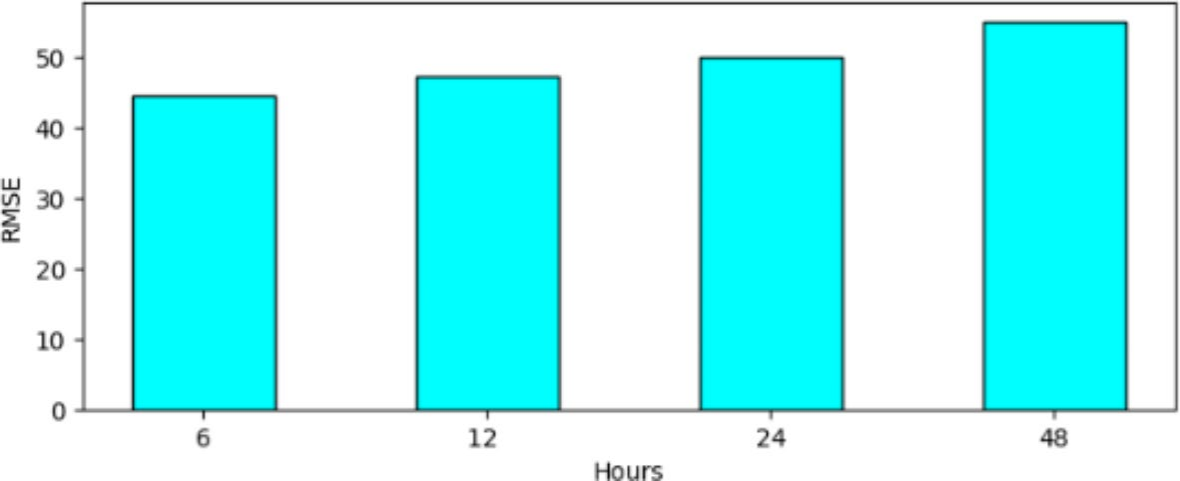
RMSE for different time interval.

The results found in Chen et al.^14^ “Air Quality Prediction Based on Integrated Dual LSTM Model,” and Al-Eidi et al.^27^ “Comparative Analysis Study for Air Quality Prediction in Smart Cities Using Regression Techniques”, Compare the performances of multiple algorithms in forecasting values of PM2.5 and PM10. Comparatively speaking the proposed TBS seems to be just as efficient in forecasting Air Quality over a generalized area with regards to the above measured metrics R2 and MAE. Figure 9 illustrates the RMSE for different time forecasts. Although the RMSE increases with the increase in the forecast duration, it does not increase significantly, indicating that the model’s performance can be effectively adapted for longer time horizons as well.

From the Metric comparison table as shown in Table 3, it is evident that the proposed TBS model has relatively high R2 scores indicating the model’s ability in identifying the variations present in the data and its accurate understanding of the underlying patterns which seems to be at a high level. The TBS dataset is more diverse compared to others models. While ARIMA alone performed well on a single-city dataset, its performance significantly declined when tested on the diverse dataset used for TBS.

**Table 3:** Comparison of TBS with other models.

Model	R-square	MAE	RMSE	MAPE
SVR^14^	0.11	39.95	55.92	72.78
Linear Regression^27^	0.79	32.19	42.70	78.86
Ridge Regression^14^	0.51	45.85	79.23	86.96
XGBoost^14^	0.78	20.21	68.24	90.97
SLSTM^28^	0.68	47.26	67.69	78.78
NLSTM^29^	0.65	37.08	47.90	73.43
Single Factor LSTM^14^	0.69	18.35	26.42	79.09
ARIMA	0.65	32.15	32.82	39.48
Multi Factor LSTM^14^	**0.81**	**12.05**	**18.77**	**42.39**
TBS (Proposed method)	**0.81**	**31.73**	**44.60**	**26.02**

Significant values are in bold.

When it comes to the error metrics namely Mean Absolute Error (MAE) and Root Mean Squared Error (RMSE) it is evident that compared to the other top performing models the TBS has a relatively higher error rate but this can be contributed to the extent of generalization that the model covers with the diversified dataset used which makes the error rates a relatively good starting point with room for improvement.

Finally in terms of Mean Absolute Percentage Error (MAPE) of 26% it is evident that the TBS model outperforms the rest of the field which makes it accurate over a larger field of data.

The proposed TBS Framework offers a comprehensive solution for AQI forecasting, leveraging spatial and temporal information. The robust metrics, coupled with visualizations, underscore the model’s effectiveness in capturing the complexities of air quality dynamics. Further exploration and validation on diverse datasets will contribute to refining the model and extending its applicability to various urban environments.

## Conclusion and future work

The proposed TBS Framework, utilizes Convolutional Neural Network (CNN) for spatial patterns and Autoregressive Integrated Moving Average (ARIMA) for temporal dependencies, demonstrates commendable performance in forecasting Air Quality Index (AQI) amidst the inherent variability in urban air quality dynamics. Enhancing the spatial network density by incorporating data from additional major cities is imperative. The current model has been trained on a dataset encompassing 22 cities, yet the gradual inclusion of more metropolitan areas into the metadata promises a more intricate and dense network. The adoption of advanced time series models represents another avenue for improvement. While the ARIMA component effectively captures temporal patterns, exploring more sophisticated algorithms. The external factors that influence air quality, such as meteorological data, industrial activities, and seasonal variations, can further refine the model’s predictive capabilities. This holistic approach would enable the model to consider a broader spectrum of influencers, thereby offering more nuanced and accurate forecasts. TBS Framework marks a promising step towards effective air quality forecasting, yet continuous research and development are essential for staying ahead of evolving urban dynamics and advancing the precision of predictions. The incorporation of more cities, adoption of advanced forecasting models, and consideration of external factors will collectively contribute to a more comprehensive and resilient air quality forecasting system, fostering sustainable urban development

## Data Availability

The air quality data used in this research was obtained from the Central Pollution Control Board of India, which provides publicly accessible reports from monitoring stations located across the country; the data set was collected from https://airquality.cpcb.gov.in/AQI_India/, which is a public link. The data compiled for this study encompasses multiple cities and regions, reflecting a diverse range of environmental conditions and air quality profiles. Researchers interested in accessing the original data for replication or further analysis can obtain it directly from the Central Pollution Control Board’s official website or through authorized channels. For assistance or clarification regarding the dataset, the first author can be contacted at shrikar.jayaraman2020@vitstudent.ac.in upon reasonable request.
